# Transcriptional Profiling Identifies Prognostic Gene Signatures for Conjunctival Extranodal Marginal Zone Lymphoma

**DOI:** 10.3390/biom13010115

**Published:** 2023-01-06

**Authors:** Julian Wolf, Thomas Reinhard, Rozina Ida Hajdu, Günther Schlunck, Claudia Auw-Haedrich, Clemens Lange

**Affiliations:** 1 Eye Center, Medical Center, Faculty of Medicine, University of Freiburg, 79106 Freiburg, Germany; 2 Molecular Surgery Laboratory, Stanford University, Palo Alto, CA 94304, USA; 3 Department of Ophthalmology, Byers Eye Institute, Stanford University, Palo Alto, CA 94304, USA; 4 Department of Ophthalmology, Semmelweis University, 1085 Budapest, Hungary; 5 Ophtha-Lab, Department of Ophthalmology, St. Franziskus Hospital, 48145 Münster, Germany

**Keywords:** conjunctival lymphoma, EMZL, prognosis, recurrence, cellular tumor microenvironment, RNA sequencing, formalin-fixation and paraffin-embedding (FFPE)

## Abstract

This study characterizes the transcriptional profile and the cellular tumor microenvironment of conjunctival extranodal marginal zone lymphoma (EMZL) and identifies prognostically relevant biomarkers. Ten formalin-fixed and paraffin-embedded conjunctival EMZL and eight healthy conjunctival specimens were analyzed by Massive Analysis of cDNA Ends (MACE) RNA sequencing. The 3417 upregulated genes in conjunctival EMZL were involved in processes such as B cell proliferation and Rac protein signaling, whereas the 1188 downregulated genes contributed most significantly to oxidative phosphorylation and UV protection. The tumor microenvironment, as determined by deconvolution analysis, was mainly composed of multiple B cell subtypes which reflects the tumor’s B cell lineage. However, several T cell types, including T helper 2 cells and regulatory T cells, as well as innate immune cell types, such as anti-inflammatory macrophages and plasmacytoid dendritic cells, were also strongly enriched in conjunctival EMZL. A 13-biomarker prognostic panel, including *S100A8* and *S100A9*, classified ocular and extraocular tumor recurrence, exceeded prognostic accuracy of Ann Arbor and American Joint Committee on Cancer (AJCC) staging, and demonstrated prognostic value for patient survival in 21 different cancer types in a database of 12,332 tumor patients. These findings may lead to new options of targeted therapy and may improve prognostic prediction for conjunctival EMZL.

## 1. Introduction

Conjunctival lymphoma is the third most frequent primary malignancy of the ocular surface with about 80% being low-grade marginal zone B-cell lymphoma [1,2]. With an incidence of about 0.2 per 100,000, conjunctival lymphoma is relatively rare [3,4]. However, the incidence has increased during recent years, and up to 20% of patients develop potentially life-threatening systemic disease [5,6]. The etiology remains poorly understood; however, factors such as reactive lymphoid hyperplasia, immune deficiency, chronic infectious agents (e.g. *Helicobacter pylori* and *Chlamydia psittaci*), autoimmune conditions, ultraviolet light, and genetic mutations have been implicated in the pathogenesis of the disease [1,3,7,8,9,10]. Since clinical differentiation from benign conjunctival lesions such as reactive lymphoid hyperplasia is challenging, incisional biopsy and histopathological and cytological examination are the gold standard for diagnosis and subtyping of conjunctival lymphoma [1]. Prognostic risk factors include Ann Arbor stage higher than stage I, higher stage according to the American Joint Committee on Cancer staging, age older than 60 years, increased expression of markers such as Ki67, and localization in the fornix or mid-bulbar conjunctiva [1,4,5,6,11]. Currently, little is known about the transcriptional profile and the cellular tumor microenvironment of conjunctival lymphoma, and no molecular biomarker panel is available to assess prognosis of this extranodal lymphoma. In this study, we apply RNA sequencing technology to characterize the transcriptional profile of conjunctival extranodal marginal zone lymphoma (EMZL) and to analyze its cellular tumor microenvironment. Thus, this study provides new insights into the pathways, molecular mechanisms, and cell types involved in disease pathogenesis, which may contribute to the definition of potentially new diagnostic and therapeutic targets as well as prognostically relevant biomarkers in the future.

## 2. Methods

### 2.1. Patients and Clinical Outcome

Ten conjunctival EMZL samples from 10 eyes of 9 patients who underwent tumor resection at the Eye Center of the University of Freiburg between 1996 and 2020 were retrospectively included in this study. Eight healthy conjunctival samples from eight patients undergoing retinal detachment surgery between 2013 and 2016 served as controls. All methods were in accordance with relevant guidelines and regulations and informed consent was obtained from all subjects. Ethics approval was granted by the Ethics Committee of the Albert-Ludwigs-University Freiburg (approval number 21-1246).

### 2.2. Formalin Fixation and Paraffin Embedding

Formalin fixation and paraffin embedding (FFPE) of tissue samples was performed immediately after surgery according to routine protocols, as previously described [12,13]. Briefly, samples were fixed immediately after surgery in 4% formalin (Carl Roth GmbH, Karlsruhe, Germany) for 12 h, dehydrated in alcohol, and processed for paraffin embedding. Diagnoses were made based on histopathological, immunohistochemical, and molecular genetic examination by at least two experienced (ophthalmic) pathologists.

### 2.3. RNA Isolation

Fifteen FFPE sections of 4 μm thickness from each sample were stored in tubes prior to RNA extraction, as previously described [12,13]. Briefly, total RNA was isolated from FFPE samples using the Quick-RNA FFPE Kit (Zymo Research, Irvine, CA, USA). Following a DNAse I digestion using the Baseline-ZERO kit (Epicentre, Illumina, San Diego, CA, USA), the RNA concentration was measured with the Qubit RNA HS Assay Kit (Life Technologies, Carlsbad, CA, USA) on a Qubit Fluorometer (Life Technologies, Carlsbad, CA, USA). The RNA quality was determined with the RNA Pico Sensitivity Assay (PerkinElmer, Waltham, MA, USA) on a LabChip GXII Touch (PerkinElmer, Waltham, MA, USA).

### 2.4. RNA Sequencing

RNA sequencing was performed using massive analysis of cDNA ends (MACE), a 3’ RNA sequencing method, as previously described [14,15]. We recently demonstrated that MACE allows sequencing of FFPE samples with high accuracy, even after more than 10 years of storage [16]. Briefly, 18 barcoded libraries comprising unique molecule identifiers were sequenced on the NextSeq 500 (Illumina, San Diego, CA, USA) with 1 × 75 bp. PCR bias was removed using unique molecular identifiers.

### 2.5. Bioinformatics

Sequencing data (fastq files) were uploaded to and analyzed on the Galaxy web platform (usegalaxy.eu) [17], as previously described [18,19,20]. Quality control was performed with FastQC Galaxy Version 0.72 (http://www.bioinformatics.babraham.ac.uk/projects/fastqc/, last access on 21 August 2021). Reads were mapped to the human reference genome (Gencode, release 38, hg38, https://www.gencodegenes.org/human/release_38.html, access on 21 August 2021) with *RNA STAR* Galaxy Version 2.7.8a [21] with default parameters using the Gencode annotation file (Gencode, release 38, https://www.gencodegenes.org/human/release_38.html, access on 21 August 2021). Reads mapped to the human reference genome were counted using featureCounts Galaxy Version 2.0.1 [22] with default parameters using the aforementioned annotation file. The output of featureCounts was imported to RStudio (version 2022.02.0+443, R version 4.1.2 http://www.rstudio.com). Gene symbols and gene types were determined based on ENSEMBL release 108 (Human genes, GRCh38.p12, https://www.ensembl.org, download on 15 November 2022) [23]. T-distributed stochastic neighbor embedding (t-SNE) was applied to assess unsupervised clustering and to check for potential batch effects [24]. Differential gene expression was analyzed using the R package DESeq2 Version 1.34.0 [25] with default parameters (Benjamini–Hochberg adjusted *p*-values). Transcripts with log2 fold change (log2 FC) > 2 or <−2 and adjusted *p*-value < 0.05 were considered as differentially expressed genes (DEG). Heatmaps were created with the R package ComplexHeatmap 2.10.0 [26]. Other data visualization was performed using the ggplot2 package [27]. Gene enrichment analysis and its visualization were done using the R package clusterProfiler 4.2.2 [28]. Cell type enrichment analysis was performed using xCell [29]. The tool uses sequencing-derived transcriptomic signatures of 64 distinct immune and stroma cell types to estimate the relative contributions of these cells to a bulk RNA transcriptome. Transcripts per million were calculated as an input for the analysis based on the output of featureCounts (assigned reads and feature length), as previously described [30]. xCell enrichment scores were compared between different groups using the Mann–Whitney U test. The log2 fold change of enrichment scores between different groups was defined as the log2 of the quotient of mean enrichment scores of each group. 

To identify a prognostic transcriptome signature of conjunctival EMZL, DEG between primary tumors which later developed a recurrence/systemic disease compared to those without recurrence were calculated. In a next step, Kaplan–Meier survival analyses were applied using the R package survival (version 3.4.0) to further specify the panel of prognostic biomarkers. Only genes were retained which had a *p*-value < 0.05 in Kaplan–Meier analysis and a gene expression which was at least two times higher in each sample with recurrence (within 4 years) compared to all samples without recurrence. Finally, a gene ontology analysis using the R package clusterProfiler 4.2.2 [28] was performed to identify biomarkers involved in the most significantly enriched biological processes in conjunctival EMZL with recurrence/systemic disease.

The association of gene expression of each biomarker with survival of patients with various cancer types was analyzed using the Gene_Outcome function of the TIMER database (http://timer.cistrome.org, access on 20 November 2022, version 2.0) [31]. The analysis is based on the transcriptional profiles and survival data from 12,332 tumors.

## 3. Results

### 3.1. Patient Characteristics

A total of 18 conjunctival samples from 18 eyes from 17 patients collected between 1996 and 2020 at our institution were included in this study. Two conjunctival lymphoma samples were obtained from both eyes of the same patient. Histological, immunohistochemical, and molecular genetic analyses confirmed conjunctival extranodal marginal zone B-Cell lymphoma (hereafter referred to as conjunctival EMZL) in ten cases and healthy conjunctiva in eight cases. Patient characteristics are summarized in Table 1. The mean ages of the conjunctival EMZL and the control group were 58.0 (range: 25.9–82.9) and 55.7 years (range: 43.0–69.0), respectively (*p* = 0.736). There were six male and four female patients in the conjunctival EMZL group and six male and two female patients in the control group (*p* = 0.867). Staging according to the Ann Arbor classification [32] was stage I (IE) in 3 (30.0%) and stage II (IIE) in 7 (70.0%) cases. TNM categories according to the American Joint Committee on Cancer (AJCC) staging manual (eighth edition) [33] were T1 in 7 (70.0%), T2 in 1 (10.0%), and T3 in 2 (20.0%) cases. During follow-up, six patients (60.0 %) developed tumor recurrence or systemic disease (three cases) after an average of 3.9 years (min: 0.3, max: 10.2 years).

### 3.2. Transcriptional Characterization of Conjunctival EMZL

**Figure 1:**
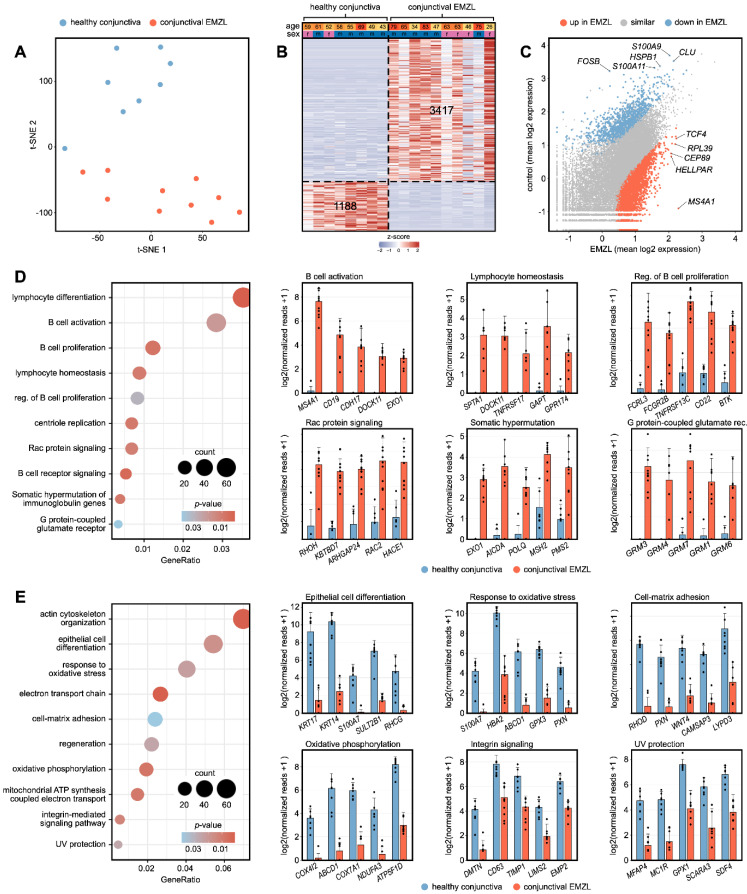
RNA sequencing reveals the transcriptional profile of conjunctival extranodal marginal zone B-Cell lymphoma (EMZL). (**A**) Unsupervised clustering using T-distributed stochastic neighbor embedding (t-SNE) analysis. Each point represents one sample. (**B**) Heatmap visualizing differentially expressed genes (DEG) between healthy conjunctiva and conjunctival EMZL (Definition of DEG: log2 FC > 2 or <−2 and adjusted *p*-value < 0.05). Basic demographic data is shown at the top. Each column represents one sample and each row one DEG. The number of DEG is given within the heatmap. The z-score represents a gene’s expression in relation to its mean expression by standard deviation units (red: upregulation, blue: downregulation). (**C**) Read plot visualizing DEG between conjunctival EMZL and healthy conjunctiva. Each point represents one gene. The top five DEG of both groups are labeled. Upregulated (**D**) and downregulated (**E**) gene ontology (GO) biological processes in conjunctival EMZL. The top ten enriched biological processes are shown in the dot plots. The size of the dots represents the number of associated genes (count). The adjusted *p*-value of each GO term is shown by color. The gene ratio describes the ratio of the count to the number of all DEG. The bar plots on the right visualize gene expression of the top five DEG involved in six of the most significantly up- or downregulated biological processes. The height of the bar represents the mean expression and the error bar corresponds to the standard deviation. Each point represents one sample. Abbreviation: EMZL: extranodal marginal zone B-Cell lymphoma.

Unsupervised cluster analysis using T-distributed stochastic neighbor embedding (t-SNE) [24] revealed distinct differences between the transcriptional profile of conjunctival EMZL and normal conjunctiva (Figure 1A). Differential gene expression analysis revealed 3417 up- and 1188 downregulated genes in conjunctival EMZL compared to healthy conjunctiva (Figure 1B). Among them, *MS4A1* (CD20, adjusted *p* = 4.8 × 10^−33^), *HELLPAR* (HELLP Associated Long Non-Coding RNA, adjusted *p* = 3.9 × 10^−24^), *CEP89* (Centrosomal Protein 89, adjusted *p* = 7.4 × 10^−26^), *RPL39* (Ribosomal Protein L39, adjusted *p* = 1.7 × 10^−12^), and *TCF4* (Transcription Factor 4, adjusted *p* = 1.1 × 10^−32^) were the five most significantly upregulated genes in conjunctival EMZL (Figure 1C). *CLU* (Clusterin, adjusted *p* = 3.0 × 10^−17^), *S100A9* (S100 Calcium Binding Protein A9, adjusted *p* = 7.0 × 10^−10^)*, HSPB1* (Heat Shock Protein Family B (Small) Member 1, adjusted *p* = 1.7 × 10^−16^), *S100A11* (S100 Calcium Binding Protein A11, adjusted *p* = 9.0 × 10^−25^), and *FOSB* (FosB Proto-Oncogene, AP-1 Transcription Factor Subunit, adjusted *p* = 1.7 × 10^−22^) were the top five downregulated genes in conjunctival EMZL (Figure 1C). Gene ontology (GO) analysis revealed, that the upregulated genes contributed most significantly to biological processes such as B cell activation (e.g., *MS4A1* (CD20) and *CD19* (CD19 molecule), lymphocyte homeostasis (e.g., *SPTA1* (Spectrin Alpha, Erythrocytic 1), and *DOCK11* (Dedicator Of Cytokinesis 11)), regulation of B cell proliferation (e.g., *FCRL3* (Fc Receptor Like 3) and *FCGR2B* (Fc Gamma Receptor IIb)), Rac protein signaling (e.g., *RHOH* (Ras Homolog Family Member H) and *KBTBD7* (Kelch Repeat And BTB Domain Containing 7)), somatic hypermutation of immunoglobulin genes (e.g., *EXO1* (Exonuclease 1) and *AICDA* (Activation Induced Cytidine Deaminase)), and G protein-coupled glutamate receptor signaling (e.g., *GRM3* and *GRM4* (Glutamate Metabotropic Receptor 3 and 4) (Figure 1D). The downregulated genes were most significantly involved in biological processes such as epithelial cell differentiation (e.g., *KRT17* (keratin 17) and *KRT14* (keratin 14), response to oxidative stress (e.g., *S100A7* (S100 Calcium Binding Protein A7) and *HBA2* (Hemoglobin Subunit Alpha 2)), cell-matrix adhesion (e.g., *RHOD* (Ras Homolog Family Member D) and *PXN* (Paxillin)), oxidative phosphorylation (e.g., *COX4I2* (Cytochrome C Oxidase Subunit 4I2) and *ABCD1* (ATP Binding Cassette Subfamily D Member 1), integrin signaling (e.g., *DMTN* (Dematin Actin Binding Protein) and *CD63* (CD63 molecule)), and UV protection (e.g., *MFAP4* (Microfibril Associated Protein 4) and *MC1R* (Melanocortin 1 Receptor) (Figure 1E). These results demonstrate distinct differences in the transcriptional profile between conjunctival EMZL and healthy conjunctiva and reveal the biological processes associated with the underlying disease.

### 3.3. Cellular Tumor Microenvironment of Conjunctival EMZL

**Figure 2:**
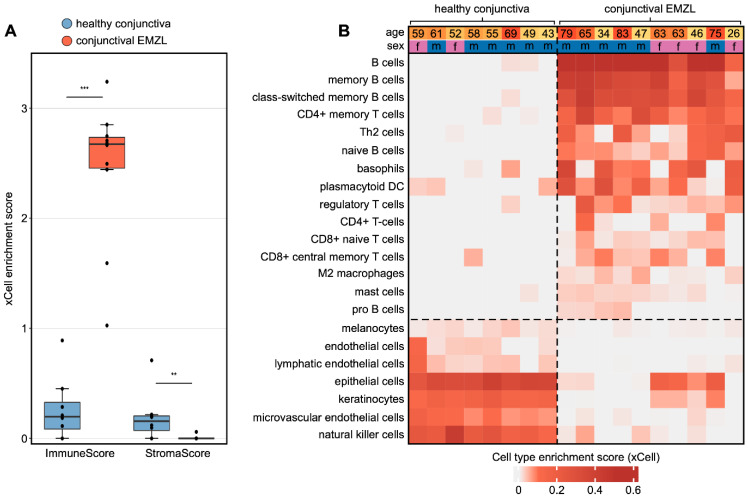
Cell type deconvolution analysis provides new insights into the tumor microenvironment of conjunctival extranodal marginal zone B-Cell lymphoma (EMZL). (**A**) Cell type enrichment analysis using xCell. The tool uses gene expression profiles of 64 immune and stromal cell types to calculate cell type enrichment scores. The boxplots visualize summary scores for immune and stroma cell types. ** *p* < 0.01, *** *p* < 0.001. Each point represents one sample. (**B**) Heatmap illustrating xCell enrichment scores of cell types which differed significantly between conjunctival EMZL and healthy conjunctiva (*p* < 0.05, Mann–Whitney U test). Each row represents one cell type, each column represents one sample. Rows are ordered according to the fold change of mean enrichment scores. Basic demographic data are shown above. Abbreviations: Th2: type 2 T-helper cells, DC: dendritic cells, EMZL: extranodal marginal zone B-Cell lymphoma.

Cell type deconvolution analysis using xCell [29] revealed significant differences in the cellular composition of conjunctival EMZL compared to healthy conjunctiva. In general, conjunctival EMZL was characterized by a substantial increase of immune cells and a decrease of stromal cells, as demonstrated by a significant increase of the ImmuneScore and a decrease of the StromaScore, both of which summarize enrichment of immune and stroma cell types, respectively (Figure 2A). When analyzing individual cell types, we found that the cellular tumor microenvironment of conjunctival EMZL was predominantly composed of multiple subtypes of B cells, which is consistent with the fact that conjunctival EMZL is a B-cell lymphoma (Figure 2B). Interestingly, the analysis also revealed a significant enrichment of various T cell types, such as CD4+ (memory) T cells, T helper 2 cells, and regulatory T cells. In addition, the tumor microenvironment included several innate immune cell types in higher quantities than in healthy conjunctiva, among them anti-inflammatory M2 macrophages, plasmacytoid dendritic cells, basophils, and mast cells (Figure 2B). At the same time, several typical cell types of healthy conjunctival tissue occurred less frequently in conjunctival EMZL, among them keratinocytes, melanocytes, and endothelial cells, further highlighting the alterations of the cellular microenvironment in conjunctival EMZL (Figure 2B).

### 3.4. Biomarkers Associated with Recurrence of Conjunctival EMZL

**Figure 3:**
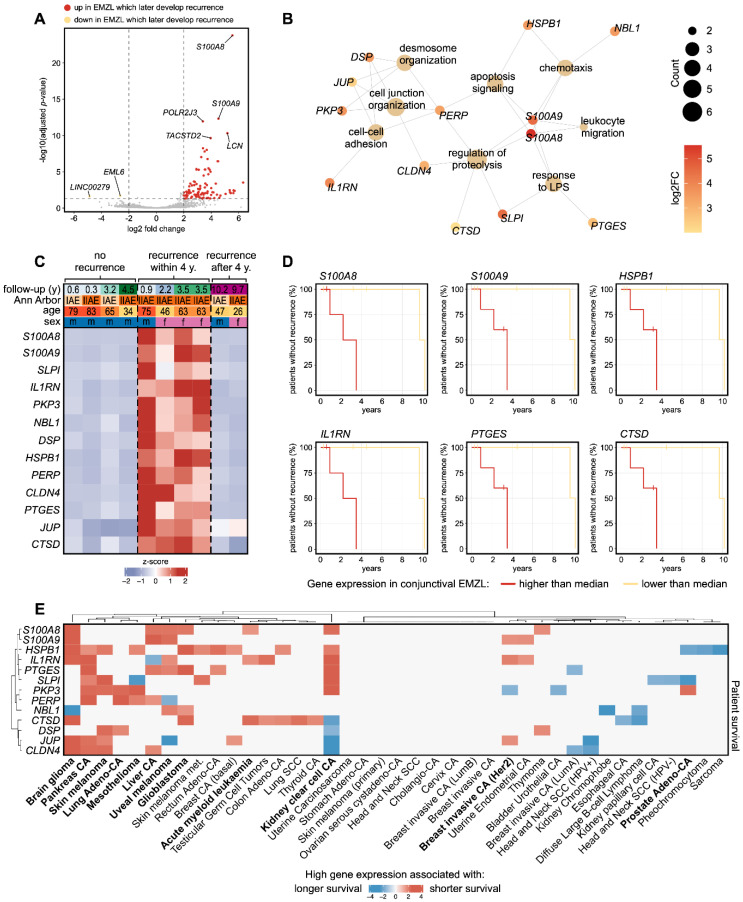
RNA sequencing identifies prognostic transcriptome signature associated with recurrence of conjunctival extranodal marginal zone B-Cell lymphoma (EMZL). (**A**) Volcano plot visualizing genes which were significantly up- or downregulated in primary conjunctival EMZL which later developed tumor recurrence/systemic disease. Genes not differentially expressed are shown in grey. Each point represents one gene. The top genes are labeled. (**B**) A gene ontology enrichment analysis was performed to identify enriched biological processes based on the identified prognostic genes. The results are shown as a network diagram. Each enriched biological process is visualized by a large circle (see label) and the associated genes (smaller points) are connected by lines. Color indicates log2 fold change between conjunctival EMZL which later develop recurrence/systemic disease and those which did not develop recurrence (see legend). The count represents the number of associated genes for each biological process. (**C**) Heatmap visualizing expression of 13 prognostic marker genes for conjunctival EMZL. Each row represents one gene and each column one tumor. The z-score represents a gene’s expression in relation to its mean expression by standard deviation units (red: upregulation, blue: downregulation). The genes are ordered according to their foldchange between poor and good prognosis. Basic demographic data as well as the Ann Arbor stage and the follow-up time are shown at the top. Tumors without recurrence are shown in the left columns, tumors which developed recurrence/systemic disease within 4 years are shown in the middle, and tumors where EMZL recurred after almost 10 years are shown on the right. (**D**) Kaplan–Meier plots visualizing rate of recurrence for samples with gene expression above (red) and below (orange) median expression. Censored patients are indicated by small vertical lines. (**E**) Prognostic biomarker are associated with patient survival in multiple cancer types. Heatmap visualizing the association of gene expression of each marker with survival of patients with various cancer types (TIMER database). A positive z-score corresponds to shorter survival with higher gene expression. The analysis is based on the transcriptional profiles from 12,332 tumors. Tumor types for which at least three of the identified biomarkers were also associated with patient survival are written in bold. Abbreviations: CA: carcinoma, HPV: Human papillomavirus, SCC: squamous cell carcinoma, EMZL: extranodal marginal zone B-Cell lymphoma.

After tumor excision, the patients in this study were followed up for an average of 3.9 years (range: 0.3–10.2). During this time, 6 of the 10 patients developed a recurrent tumor. This follow-up data allowed us to exploratively search for biomarkers in the gene expression profile of the primary tumor with potential prognostic relevance for tumor recurrence. In the first step, differentially expressed genes (DEG) between primary tumors which later developed a recurrence or systemic disease compared to those without recurrence were calculated (Figure 3A) and Kaplan–Meier analyses were performed for each DEG to further specify the biomarker panel. A pathway enrichment analysis revealed the most significantly enriched biological processes these DEG were involved in, among them apoptosis signaling, chemotaxis, regulation of proteolysis, and cell–cell adhesion (Figure 3B). This analysis identified 13 key biomarkers with prognostic relevance for tumor recurrence/systemic disease within 4 years of tumor excision, among them *S100A8* (S100 calcium binding protein A8), *S100A9* (S100 calcium binding protein A9), *HSPB1* (Heat Shock Protein Family B (Small) Member 1), *IL1RN* (Interleukin 1 Receptor Antagonist), *PTGES* (Prostaglandin E Synthase), and *CTSD* (Cathepsin D) (Figure 3C,D). However, the biomarkers were only able to predict recurrence/systemic disease in aggressive tumors which were associated with early tumor recurrence within 4 years after tumor excision, but not for the two patients in which recurrence occurred after 9.7 and 10.2 years (Figure 3C). 

Having identified 13 prognostic biomarkers for recurrence in conjunctival EMZL, we next explored whether they were also associated to patient survival in various other cancer types. For this analysis, we used the TIMER database [31], which provides the transcriptional profiles and survival data for 12,332 tumor patients. This analysis revealed that increased gene expression of all 13 biomarkers was associated with shorter patient survival in 21 different cancer types, among them pancreas carcinoma, skin melanoma, glioblastoma, acute myeloid leukemia, kidney clear cell carcinoma, and others (Figure 3E). 

Finally, we investigated whether clinical or histopathological parameters, such as age, sex, Ann Arbor staging, and AJCC staging were associated with clinical outcome (Table 2). Strikingly, the clinical and histopathological parameters assessed were not significant in Kaplan–Meier analysis, whereas the transcriptome signature was significantly associated with tumor recurrence/systemic disease (*p* = 0.014).

Taken together, this study exploratively identifies 13 biomarkers which are associated with ocular and extraocular tumor recurrence of conjunctival EMZL exceeding prognostic accuracy of Ann Arbor and AJCC staging. These findings may contribute to improved prediction of tumor recurrence of conjunctival EMZL, but require further validation in prospective studies with larger sample sizes.

## 4. Discussion

Transcriptional profiling has provided new insights into the molecular mechanisms of tumor formation and progression and has helped to define new therapeutic targets in numerous malignancies [12,15,34]. However, RNA sequencing of rare tumors, such as conjunctival EMZL, has so far been challenged by their low incidence. Specialized 3′-RNA sequencing methods such as Massive Analysis of cDNA Ends (MACE) enables transcriptional profiling of formalin-fixed and paraffin-embedded (FFPE) conjunctival tissue with high accuracy even after more than 10 years of storage [16]. Here, we apply MACE RNA sequencing to FFPE specimens to characterize the transcriptional profile and the cellular tumor microenvironment of conjunctival EMZL compared to healthy conjunctiva. By integrating this data with the clinical follow-up of each patient, we further identify a prognostic biomarker panel which may contribute to improved prediction of tumor recurrence/systemic disease of conjunctival EMZL. 

The gene expression profile of conjunctival EMZL differed significantly from healthy conjunctival specimens and revealed 3417 up- and 1188 downregulated genes. Gene ontology (GO) analysis demonstrated that the upregulated genes were mainly involved in biological processes such as regulation of B cell proliferation, Rac protein signaling, and G protein-coupled glutamate receptor signaling. *FCGR2B* (Fc Gamma Receptor IIb) was among the top upregulated genes involved in regulation of B cell proliferation. Expression of *FCGR2B* has been shown to decrease the response rate to rituximab monotherapy in follicular lymphoma [35]. Rituximab is a monoclonal antibody against CD20 and also one of the well-established therapeutic strategies in conjunctival B-cell lymphoma [1]. Considering the highly variable *FCGR2B* expression which we observed in our study, *FCGR2B* may be a potential biomarker to guide therapeutic intervention in conjunctival EMZL. However, this question needs to be further investigated in future studies. *KBTBD7* (Kelch Repeat and BTB Domain Containing 7) and *RAC2* (Rac Family Small GTPase 2) were among the top upregulated genes in Rac protein signaling. Downregulation of *KBTBD7* was demonstrated to inhibit cell proliferation and invasion of non-small cell lung cancer cells in vitro [36] and small interfering RNA-mediated knockdown of *RAC2* demonstrated antiproliferative and proapoptotic effects in prostate cancer cells [37]. These results indicate that both *KBTBD7* and *RAC2* may represent potential new therapeutic targets for conjunctival EMZL. *AICDA* (Activation Induced Cytidine Deaminase) was another top overexpressed gene in conjunctival EMZL. The gene has been reported to be highly expressed in B-cell-derived malignancies and RNA interference-mediated knock-down has been shown to slow down lymphoma cell proliferation [38]. The upregulated genes were also enriched in G protein-coupled glutamate receptor signaling, among them *GRM3* and *GRM4* (Glutamate Metabotropic Receptor 3 and 4). *GRM3* plays important roles in colon cancer pathogenesis and knockdown of *GRM3* in colon cancer cells reduced tumor cell survival in vitro and inhibited tumor growth in vivo [39]. The specific expression of *GRM3* in conjunctival EMZL without any relevant expression in healthy conjunctiva further highlights *GRM3* as a potential therapeutic target for conjunctival EMZL. The downregulated genes were most significantly involved in processes such as oxidative phosphorylation, integrin signaling, and UV protection. The downregulation of oxidative phosphorylation may indicate that the Warburg effect—a metabolic deregulation which occurs in many cancer types and is characterized by increased aerobic glycolysis and impaired oxidative phosphorylation—may also be involved in conjunctival EMZL [40]. *DMTN* (Dematin Actin Binding Protein), one of the top downregulated genes, was recently identified as a vital tumor suppressor in glioblastoma multiforme, which significantly suppressed tumor growth in vivo [41]. In addition, UV protection was one of the downregulated pathways which is consistent with ultraviolet light being discussed as a risk factor for conjunctival EMZL [1]. One of the involved genes was *MFAP4* (Microfibril Associated Protein 4), which plays an essential role in photoprotection of the skin. Its downregulation observed in conjunctival EMZL may increase the susceptibility of the conjunctiva to ultraviolet light [42]. These findings reveal the biological processes and key genes which are modulated in conjunctival EMZL. Further studies will be necessary to investigate the presented factors and signaling pathways in more detail and to validate them as potential therapeutic targets for the treatment of conjunctival EMZL.

The cellular tumor microenvironment is known to modulate tumor progression, therapeutic response, and clinical outcome of various malignancies [43]. Here, we applied transcriptome-based cell type deconvolution analysis using xCell [29] revealing that the tumor microenvironment of conjunctival EMZL was predominantly composed of multiple subtypes of B cells which reflects the tumor’s B cell lineage. Interestingly, we also found that several T cell types were strongly enriched, among them T helper 2 cells and regulatory T cells. T helper 2 cells have been shown to induce B cell proliferation and to be essential for the development and progression of low-grade B-cell lymphoma [44,45,46]. In addition, several innate immune cell types were enriched in conjunctival EMZL, among them anti-inflammatory macrophages, plasmacytoid dendritic cells, and mast cells. Anti-inflammatory macrophages represent critical immune cells in the tumor microenvironment and are able to promote lymphoma growth and can impair the patient’s prognosis [47]. Plasmacytoid dendritic cells are known to be important components of the tumor microenvironment in various malignancies and are essentially involved in the regulation of anti-tumor immunity [48,49]. Finally, mast cell infiltration has been observed in various tumors, including skin melanoma, breast, and colorectal cancer [50]. Mast cells are able to modulate the tumor microenvironment by releasing different mediators, including a variety of proangiogenic factors and several matrix metalloproteinases that can increase the invasiveness of the tumor [50]. Taken together, these findings suggest that apart from B cells, several other immune cell types are enriched in the tumor microenvironment of conjunctival EMZL and that they may represent potential therapeutic targets for a specific anti-tumor immune response.

In search of a prognostic tool that provides information on the risk of recurrence/systemic disease of conjunctival EMZL, this study identified a prognostic biomarker panel comprising 13 genes which allows us to classify for recurrence within 4 years after excision of the primary tumor. Analyzing the transcriptional profiles and survival data from another 12,332 tumor patients from the TIMER database [31], we found that increased expression of all 13 identified biomarkers was associated with shorter patient survival in 21 different cancer types, including pancreas carcinoma, skin melanoma, glioblastoma, acute myeloid leukemia, and kidney clear cell carcinoma. In contrast, established predictors of poor prognosis in conjunctival EMZL such as Ann Arbor and AJCC stage were not significantly associated with the clinical outcome in our study, although some tendency was observed, which may be explained by the low sample size of our study. In line with these findings, other studies also revealed that AJCC staging has limited prognostic value in conjunctival EMZL [4]. These results underline the potential of a gene expression biomarker panel to categorize clinical outcome. Our findings may improve prediction of tumor recurrence of conjunctival EMZL, but require further validation in prospective studies with larger sample sizes. In addition, our findings may represent the foundation for future studies investigating epigenetic modulations of these genes and whether the multi-omics integration of gene expression and epigenetic data can further benefit the prognostic classification of EMZL [51,52].

We acknowledge that this study is limited by its retrospective single center design and its relatively small sample size. However, a prospective single center study is challenging due to the very low incidence of conjunctival EMZL and a prospective multicenter study would be necessary to overcome this limitation. Another limitation is the lack of external validation of the prognostic biomarker panel, since no sequencing data of conjunctival EMZL are available so far. Furthermore, in contrast to single cell RNA sequencing (scRNA), bulk RNA sequencing cannot provide insights into cell heterogeneity and thus cannot reveal cell-specific gene expression profiles to discern possible subtypes of tumor cells. However, scRNA sequencing is not feasible on FFPE samples. Therefore, we employed a bulk RNA sequencing-based cell type deconvolution analysis using xCell [29], which is one of the most accurate tools available [53], to characterize the cell types enriched in the tumor microenvironment. It is important to emphasize that the cell type investigations are based on in silico analyses and have not been validated histologically due to the limited number of specimens. This needs to be addressed in future studies.

In summary, this study applies high-resolution transcriptional profiling on conjunctival EMZL providing new insights into the pathways, molecular mechanisms, and cell types involved in disease pathogenesis. In addition, a biomarker panel consisting of 13 tissue-specific factors for predicting ocular and extraocular tumor recurrence is presented. These results may lead to new options of targeted therapy and improved prognostic prediction for conjunctival EMZL.

## Figures and Tables

**Table 1:** Patient characteristics.

Group	Conjunctival EMZL	Healthy Conjunctiva	*p*
*n*	10	8	-
Age at surgery (years)	58.0 (25.9–82.9)	55.7 (43.0–69.0)	0.736
Sex			0.867
Male	6 (60.0%)	6 (75.0%)	
Female	4 (40.0%)	2 (25.0%)	
Ann arbor staging		-	-
IE	3 (30.0%)		
IIE	7 (70.0%)		
AJCC staging (8th edition)		-	-
T1	7 (70.0%)		
T2	1 (10.0%)		
T3	2 (20.0%)		
Recurrence	6 (60.0%)	-	-
Ocular	3 (30.0%)		
Systemic disease	3 (30.0%)		
Time to event (years)	5.0 (0.9–10.2)	-	-

Data is shown as mean (range) or as absolute (relative) numbers. AJCC: American Joint Committee on Cancer, EMZL: extranodal marginal zone B-Cell lymphoma.

**Table 2:** Prognostic markers in conjunctival extranodal marginal zone B-Cell lymphoma (EMZL).

Group	No Recurrence	Recurrence/ Systemic Disease	*p*-Value (Kaplan–Meier)
*n*	4	6	-
Age at surgery (years)	65.1 (33.6–82.9)	53.3 (25.9–74.9)	0.254 *
Sex			0.242
Male	4 (100.0%)	2 (33.3%)	
Female	0 (0.0%)	4 (66.7 %)	
Ann arbor staging			0.126
IE	2 (50.0%)	1 (16.7%)	
IIE	2 (50.0%)	5 (83.3%)	
AJCC staging (8th edition)			0.746
T1	3 (75.0%)	4 (66.7%)	
T2	1 (25.0%)	0 (0.0%)	
T3	0 (0.0%)	2 (33.3%)	
Transcriptome signature			0.014
no recurrence	4 (100.0%)	2 (33.3%)	
recurrence/systemic disease	0 (0.0%)	4 (66.7%)	

Comparison of established predictors and the identified predictive gene expression biomarker panel regarding the clinical outcome of conjunctival EMZL. Data is shown as mean (range) or as absolute (relative) numbers. The *p*-values are calculated with Kaplan–Meier analyses. * Age was categorized based on median age in old (>63.4 years) and young (≤63.4 years) patients. AJCC: American Joint Committee on Cancer, EMZL: extranodal marginal zone B-Cell lymphoma.

## Data Availability

The sequencing data are available in the Gene Expression Omnibus Database (https://www.ncbi.nlm.nih.gov/geo/) under the accession number GSE218618.
